# Paramedian tuberculum sellae meningioma: tumor resection and optic canal unroofing via contralateral supraorbital eyebrow approach

**DOI:** 10.3171/2025.10.FOCVID25166

**Published:** 2026-01-01

**Authors:** Ravi Ranjan, Kuntal Kanti Das, Sudarsana Gogoi, Sudhakar Madheshiya, Arun Kumar Srivastava

**Affiliations:** 1 Department of Neurosurgery, Sanjay Gandhi Postgraduate Institute of Medical Sciences, Lucknow; and; 2 Department of Pathology, Era’s Lucknow Medical College, Lucknow, Uttar Pradesh, India

**Keywords:** unilateral transcranial approach, optic canal unroofing, olfaction, anterior clinoidectomy

## Abstract

Tuberculum sellae meningioma (TSM) causes disproportionate vision loss due often to an associated bony hypertrophy or an intradural tumor invasion into the optic canals. Several surgical approaches are available for resecting the TSMs. A contralateral transcranial approach from the side of better/normal vision may be beneficial in asymmetrical TSMs. It allows a favorable angle of attack and access to the medial wall of the affected optic canal. Here, the authors demonstrate the technique of a contralateral supraorbital eyebrow approach (c-SOA) that could be useful in a specific subset of TSMs with good oncological and cosmetic outcome.

The video can be found here: https://stream.cadmore.media/r10.3171/2025.10.FOCVID25166

## Transcript

### 0:20 Introduction.

In this video the authors present paramedian tuberculum sellae meningioma tumor resection and optic canal unroofing via a contralateral supraorbital approach. There are no disclosures to be made. Tuberculum sellae meningiomas arise from the tuberculum sella, the limbus sphenoidale, and the chiasmatic sulcus,^1,2^ and they cause visual symptoms because of pressure or sometimes tumor extension into the optic canals and optic canal hypertrophy.^3^ There are several approaches available,^2,4,5^ and in some cases a contralateral transcranial approach may be advisable, especially if the tumor is asymmetrical and is causing ipsilateral optic nerve compression.^6–8^ In this video we show a case of contralateral supraorbital approach to an asymmetrical tuberculum sellae meningioma.

### 1:02 Case Summary.

This patient was 34-year-old lady who presented with irregular menstrual cycles for 6 months, painless progressive vision loss on the left side for 4 months, and headache for 4 months. On examination, the vision on the left side was 3/60 with left-sided optic atrophy, whereas vision was 6/12 on right side. There were no other neurological deficits or endocrinological deficits.

### 1:26 Preoperative Imaging.

Imaging shows an asymmetrical suprasellar lesion located on the left side, medial to the optic nerve, with possible medial optic canal extension. The epicenter is on the left side, and we can see that the tumor is located in the sagittal section in the tuberculum sellae area.

### 1:42 Operative Plan.

We planned a contralateral supraorbital approach because it would give us direct vision of the tumor. It will also give us direct access to the medial optic canal and a right-sided approach; a contralateral approach here would be from the nondominant side. Therefore we took this patient for a contralateral supraorbital eyebrow approach.

### 2:01 Position.

This was its position. The head was rotated by around 10°–15° to the opposite side and slightly extended. We can see the marking of a lateral two-thirds eyebrow incision for a supraorbital approach.

### 2:15 Craniotomy.

We can see a supraorbital craniotomy has been done. The bony projections on the frontal skull base are drilled to make the trajectory flat.

### 2:25 Durotomy.

The dura is opened in a cruciate shape based on the frontal skull base.

### 2:32 Arachnoid Dissection and Tumor Resection.

By gradual creeping movements, the base of the brain is accessed and basal sylvian fissure is opened as it will let out some CSF; the brain becomes lax and the working space expands. Meticulous and sharp dissection of the arachnoid band is essential to expose the structures. Here we can see the ipsilateral internal carotid artery. We see the first look of the tumor and the ipsilateral, that is right-sided, optic nerve. We can see the distance between the two. This is the opposite side, the left side.

The base of the tumor is coagulated and divided. The tumor here can be seen as soft and moderately vascular. With ultrasonic aspiration, the tumor is decompressed, and gradually we do the arachnoid dissection and a layer of arachnoid is left over the optic nerve, optic chiasm, which can be seen here. Preservation of this arachnoid is very important to preserve the blood supply to the optic apparatus. The base of the tumor is further divided until we were able to see the important structure that is the left-sided optic nerve. Beneath here, the left-sided optic nerve is seen and this is traditionally a blind spot of an anterolateral approach. The medial side of the optic nerve and in a contralateral approach like this, we get a good view of this area. After debulking the tumor, it is very easy to roll the tumor and dissect the arachnoid. Preservation of this arachnoid is very, very important. Here we see the tumor is being gently rolled off. We can see the left optic nerve very well. Now the bulk of the tumor has been removed and we can see a portion of the tumor, which is where the tumor was based, just medial to the left optic nerve. This part of the tumor is gently dissected and brought into the surgical field and removed. We can see the optic nerve. We can see the interoptic space. We can see the Liliequist membrane here. Small tumor remnant is still visible and it is very important to remove that by gradual, gentle and teasing movement with angled instruments like angle dissector that is being used here.

### 5:24 Medial Optic Canal Unroofing.

Now, we add a medial optic canal unroofing. This is the advantage of this approach: one can access the medial part of the contralateral optic canal under direct vision. Using a diamond burr, the optic canal is opened, and with upcutting rongeurs, the opening is further widened. It also gives us an opportunity to resect the involved infiltrated dura. Now canal opening has given us access to this medial part of the tumor, which could otherwise not be accessed unless you open the optic canal. The tumor is gently removed and once all the tumor is removed, the dural base is coagulated with angled bipolar forceps. The bony hypertrophy of the tuberculum sella is visible and it is drilled as much as possible, taking due care to prevent injury to the adjoining areas. This is at the end. We can see that the tumor has been resected, the medial optic canal has been drilled. The involved optic nerve is quite free and looks healthy. We don’t see any tumor inside the optic canal anymore. We can see the optic chiasm covered with a shining arachnoid. We can see pituitary stalk and arachnoid covering it.

### 6:58 Hemostasis and Closure.

Hemostasis was obtained and following this, closure was performed by replacing the bone flap back.

### 7:03 Postoperative Clinical Outcome.

Postoperatively, the patient did not have any new-onset endocrinological or neurological deficit. At follow-up, the vision is improved to 6/60 and on the right side it remained same. The patient remains functionally independent. The follow-up clinical photograph shows a good cosmetic outcome and good frontalis function.

### 7:21 Postoperative Radiological Outcome.

The postoperative imaging shows the bony defect and the medial optic canal drilling that was performed. And MRI shows complete tumor resection, and we don’t see any collateral damage on the frontal lobes.

### 7:32 References.

So these are the references, and thank you for watching this video.

## Disclosures

The authors report no conflict of interest concerning the materials or methods used in this study or the findings specified in this publication.

## Author Contributions

Primary surgeon: Das, Gogoi. Assistant surgeon: Ranjan, Madheshiya. Editing and drafting the video and abstract: Das, Ranjan, Gogoi, Madheshiya. Critically revising the work: Das, Gogoi, Srivastava. Reviewed submitted version of the work: Das, Gogoi, Srivastava. Approved the final version of the work on behalf of all authors: Das.

## Supplemental Information

### Patient Informed Consent

The necessary patient informed consent was obtained in this study.
